# Technology Acceptance of 2 mHealth Apps During an Observational Technology Evaluation Study: Qualitative Results of a Mixed Methods Study

**DOI:** 10.2196/70873

**Published:** 2026-04-29

**Authors:** Johanna Graeber, Elke Warmerdam, Svenja Aufenberg, Christopher Bull, Kristen Davies, Juliane Döhring, Kirsten Emmert, Claire Judd, Corina Maetzler, Nikolay V Manyakov, Ralf Reilmann, Wan-Fai Ng, Victoria Macrae, Walter Maetzler, Hanna Kaduszkiewicz

**Affiliations:** 1 Institute of General Practice University Medical Center Schleswig-Holstein Kiel University Kiel Germany; 2 Innovative Implant Development (Fracture Healing) Division of Surgery Saarland University Saarbrücken Germany; 3 Department of Neurology University Medical Center Schleswig-Holstein Kiel University Kiel Germany; 4 George Huntington Institute Münster Germany; 5 OpenLab School of Computing Newcastle University Newcastle upon Tyne United Kingdom; 6 Translational and Clinical Research Institute Newcastle University Newcastle upon Tyne United Kingdom; 7 Department of Neurology University Medical Center Schleswig-Holstein, Campus Kiel Kiel University Kiel Germany; 8 Department of Health Promotion and Prevention Institute of Public Health and Nursing Research University of Bremen Bremen Germany; 9 NIHR Newcastle Clinical Research Facility Newcastle upon Tyne Hospitals NHS Foundation Trust Newcastle upon Tyne United Kingdom; 10 Johnson & Johnson Beerse Belgium; 11 Department of Clinical Radiology University of Münster Münster Germany; 12 Department of Neurodegeneration Hertie Insitute for Clinical Brain Research University of Tübingen Tübingen Germany; 13 Clinical Research Facility University College Cork Cork Ireland

**Keywords:** mobile health care, mobile health, mHealth, digital health, technology use, neurodegeneration, app, user experience, mixed method study

## Abstract

**Background:**

Mobile health (mHealth) apps are useful tools for research and disease management. However, implementation of mHealth apps is lacking in many areas. While mHealth apps offer various advantages to researchers and patients, their effectiveness depends on their actual use. Barriers to using mHealth apps are often due to human factors such as usability or technology acceptance. Although prior studies have examined the acceptance of mHealth apps in patient treatment, the key factors driving or hindering the use of mHealth apps in research remain unclear.

**Objective:**

This study explores user perceptions of 2 mHealth apps in the setting of an observational technology evaluation study using the unified theory of acceptance and use of technology. We aim to evaluate the technology acceptance of these specific apps and to investigate challenges in choosing suitable mHealth apps in research. The apps were intended for data collection; no effect on health was expected.

**Methods:**

Patients with chronic diseases as well as healthy participants used a symptom tracking app and a cognitive test app over the course of 4 weeks within the feasibility study of the project “Identifying Digital Endpoints to Assess Fatigue, Sleep and Activities of Daily Living in Neurodegenerative Disorders and Immune-Mediated Inflammatory Diseases.” Thereafter, 61 qualitative interviews were conducted, recorded, and transcribed. A qualitative content analysis using the unified theory of acceptance and use of technology was performed.

**Results:**

An important aspect of motivation for participants was feedback on their health data and performance in the cognitive tests. Effort played a significant role in app use. Patients rated the apps as easy to use and quick. Using the app multiple times per day at fixed times was perceived as disruptive. Participants preferred using their own phone. Social influence as well as facilitating conditions played a lesser role in intention to use the apps. Data security was no concern for most participants. They stressed the importance of good relations with the study team.

**Conclusions:**

In choosing suitable apps, one size will certainly not fit all. For medical research, pretesting of all materials with the potential users is of utmost importance. If the positive effects of the app on users’ health are not immediately apparent, other factors may motivate use, for example, feedback, gamification, adjustable functions, applicability on all smartphone operating systems, and good relations to the study team.

## Introduction

Technology has become increasingly important in maintaining our health and treating illness [1]. This affects not only care providers but also individuals who take advantage of technologies such as websites, smart devices, and apps. Mobile health (mHealth) apps, for example, monitor users’ health, remind themselves of their medication, and connect with other patients and their care providers [2-4]. The increase in use has led to an increase in the availability of these services [2,5-7]. mHealth apps can be useful tools for patients as well as medical staff. They can be particularly effective in the self-management of patients, collection of health data, support of diagnosis, and disease management [2-4,7,8]. In clinical research, mHealth apps deliver convenient access to large amounts of real-time health data from users’ everyday lives [2]. This combination of advantages highlights the growing significance of mHealth apps in patient care and medical research [9].

Although a large number of mHealth apps are available and their advantages seem obvious, implementation is still lacking in many areas. Barriers that prevent the implementation of mHealth apps are often due to human issues, such as user acceptance, low familiarity with technology, and resistance to change [10]. A prerequisite to the successful implementation of an mHealth app is its usability. Technology acceptance and the ability to implement the technology in daily life are further requirements [11]. The effectiveness of a new mHealth app depends on its actual use [5]. Therefore, the experiences and opinions of users and patients must be taken into account when evaluating mHealth apps. Understanding what aspects of mHealth apps facilitate or hinder user acceptance is an important step to promote their implementation.

The concept of technology acceptance was originally used in organizational contexts [12], such as the adoption of new technology in the workplace. However, its importance in health research has increased with the growing use of health technology such as mHealth apps [10]. Different models have been used to explain the intention and use of a technology [6], such as the theory of reasoned action or the technology acceptance model [12-14]. The unified theory of acceptance and use of technology (UTAUT) was developed to unify these different models [14] and has been widely used in mHealth research [6,15]. It postulates that the actual use of a technology can be predicted by the behavioral intention to use a technology. The behavioral intention is determined by 4 constructs: performance expectancy, effort expectancy, social influence, and facilitating conditions [13]. Performance expectancy is defined as the degree to which a user expects a technology to help him or her. Several studies on mHealth services find positive effects of performance expectancy on behavioral intention to use a technology [6,16-18]. Effort expectancy describes the assumed ease of use of a technology and has been strongly associated with a greater intention to use in an mHealth context, especially in older populations [6,16]. Venkatesh et al [13] defined social influence as “the degree to which a person perceives that important others believe he or she should use the new system.” Social influence also has a positive effect on behavioral intention in mHealth contexts [6,16]. Facilitating conditions describe the perceived organizational and technical infrastructure in support of using a technology, such as instructions or assistance [16].

A number of studies have been conducted on the technology acceptance of mHealth apps using the UTAUT or related models. Many of these studies focus on mHealth apps designed to help patients with self-management of their health such as hypertension or weight management apps [17,19,20]. Studies on diabetes management apps point out the importance of trust, particularly regarding data security and perceived disease threat [21,22]. Similar results were found regarding technology acceptance on COVID-19 contact tracing apps [23]. Technology acceptance of mHealth apps focusing on health information has also been investigated. Intention to use a prenatal care app has been investigated using the UTAUT [24]. Alsyouf et al [25] stressed the importance of data security on intention to use a personal health record system. However, the majority of these studies focus on mHealth apps that provide direct benefits to their users, such as support in health management or providing health information. The notion of perceived usefulness or performance expectancy is a well-described predictor of intention to use mHealth apps [6,7,18]. This influence might be diminished in the context of research-focused mHealth apps because they do not offer direct benefits for the user.

Here, we report the results of a study on the technology acceptance of 2 mHealth apps developed for research purposes. Patients with Parkinson disease, Huntington disease, rheumatoid arthritis, systemic lupus erythematosus, primary Sjögren syndrome, and inflammatory bowel disease as well as healthy participants used 2 mHealth apps over the course of 4 weeks. This study was part of a larger evaluation study with the goal to examine usability of these mHealth apps as well as other digital technologies. The symptom tracking app included questionnaires regarding the patient’s state of health, that is, sleepiness, fatigue, and mood. The cognitive test app included 3 different cognitive tests.

In short, this study serves 2 purposes. First, we assess the technology acceptance of these apps to evaluate their usability for a follow-up study. Second, we explore the specific challenges involved in selecting suitable data-gathering mHealth apps for medical research. We hope these insights will be helpful for researchers in planning future studies using mHealth apps and help prevent data loss due to low technology acceptance.

## Methods

### Study Overview

#### Study Design of the IDEA-FAST Feasibility Study

We conducted qualitative semistructured interviews with participants of the feasibility study (FS) of the IDEA-FAST project (“Identifying Digital Endpoints to Assess Fatigue, Sleep and Activities of Daily Living in Neurodegenerative Disorders and Immune-Mediated Inflammatory Diseases”). IDEA-FAST investigates whether digital parameters can be used to assess fatigue and sleep disturbances. The FS was an observational study, in which different technologies and mHealth apps were assessed for their suitability for the larger clinical observation study. The FS included 2 mHealth apps, a symptom tracking app [26] and a cognitive test app. Quantitative and qualitative measures were used to evaluate the suitability of the mHealth apps. For the qualitative evaluation, patients as well as healthy participants were interviewed after using the apps for 4 weeks.

The design and rationale of the FS as well as baseline characteristics have been described previously [27]. In short, digital technologies, including symptom tracking and cognitive test apps, were implemented alongside surveys and patient-reported outcome measures. The FS was conducted from August 2020 until June 2021 at 4 study sites: Erasmus University Medical Centre, Rotterdam (the Netherlands), Newcastle University (the United Kingdom), University Medical Centre Schleswig-Holstein, Kiel, and George Huntington Institute GmbH, Münster (Germany). Participants were introduced to the mHealth apps at a baseline visit with their local site. Afterward, participants used the apps for 4 weeks, each week consisting of a 5-day wearing period and a 2-day rest period. Taken together, the apps were used for 20 days. Additional information and support were offered by clinical staff either via support materials, tutorials, telephone, and/or in-person visits.

#### mHealth Apps

##### Overview

Both apps could be installed either on participants’ personal phones or on a study phone provided by the research staff. The symptom tracking app was available for Android operating systems, and the cognitive test app for both Android and IOS operating systems.

##### Symptom Tracking App

The symptom tracking app has been developed by VTT in accordance with the study design. It was scheduled to be filled out 4 times a day, at 9 AM, 1 PM, 5 PM, and 9 PM. Participants received notifications to answer questions and were given a time frame of several hours for each action within the app. Each action consisted of several questions specially designed for the study regarding current fatigue, sleepiness, mood, and pain. In the morning, users answered additional questions regarding sleep duration, sleep disturbances, and sleep quality. In the evening, physical and mental activity during the day was assessed. In addition, the study team decided to add a voice feature to allow participants to verbally share information about their day. Furthermore, 2 speech tests were included in the app for study purposes: an oral motor skill task, in which participants had to make certain sounds; and a temporal orientation task, where participants stated the time of day. The app is equipped with a reminder function using sounds, which can be turned off.

##### Cognitive Test App

**Figure 1:**
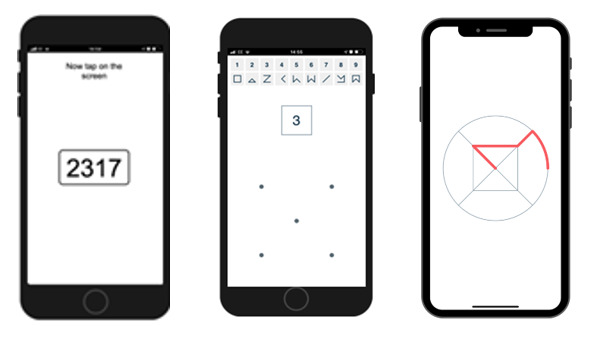
The 3 tests of the cognitive test app.

The cognitive test app has been developed by Cambridge Cognition, also in accordance with the study design. It required participants to complete a cognitive task twice a day. The psychomotor vigilance task (PVT) was administered in the first week, followed by the digit symbol substitution test (DSST) in the second week, and the n-back task (NBX) during weeks 3 and 4 [28]. The tests can be seen in Figure 1. The PVT measures reaction time, alertness, and attention. As participants look at the screen of their smartphone, a number appears, and as soon as they see the number, they have to touch the screen as quickly as possible. The DSST measures cognitive ability and takes 1.5 minutes. Participants see abstract symbols associated with numbers at the screen of their smartphone. When a particular number appears on the screen, they have to draw the corresponding symbol. Participants are asked to draw as many symbols as possible. The NBX measures attention and working memory and takes up to 2 minutes. Participants see a stream of abstract symbols. They must touch the screen whenever a visible symbol matches the one that appeared 2 symbols previously [29].

### Qualitative Interviews

**Figure 2:**
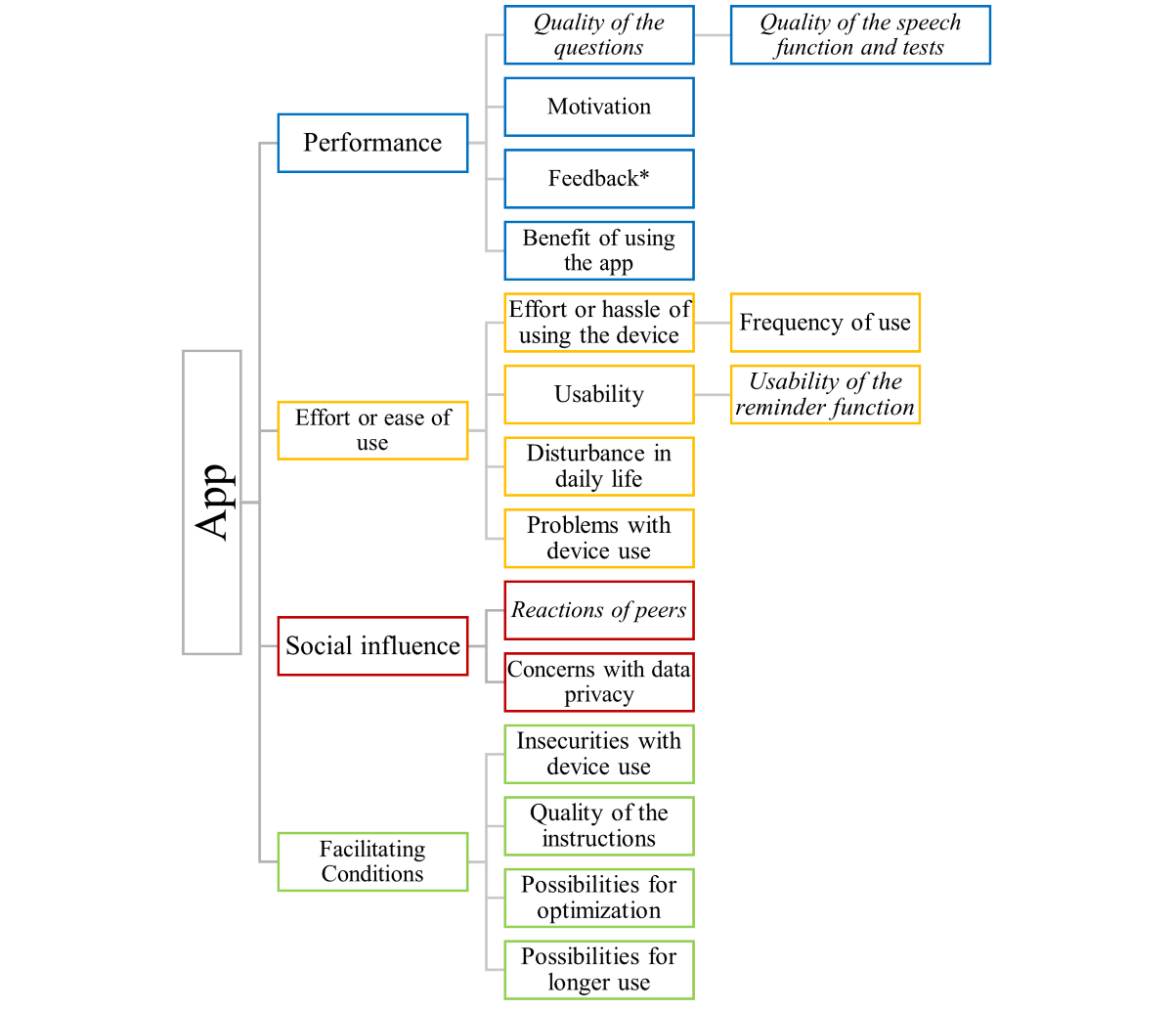
General code system of the apps. Codes present in italics format are only used for the symptom tracking app, and feedback* is only used for the cognitive test app.

The general implementation and transcription procedures of the interviews have been described previously [27]. In short, an interview guideline based on the UTAUT model was developed. After an introduction including verbal consent, some basic demographic questions followed. Participants were asked about each app separately. First, participants were asked to describe their general experiences with the app, followed by more detailed questions to cover all aspects of UTAUT. Participants in the FS were contacted during the study to assess their willingness to take part in an interview. Interviews were conducted between October 2020 and June 2021, mostly via videoconference. Written consent to participate in the interviews was obtained beforehand. Interviews were conducted in the first language of the participants and recorded with their consent. They were transcribed verbatim or pseudonymized by a transcription service. Interviews in Dutch were translated into English with an online translation tool and double-checked by a native speaker. Quotations used in this paper were slightly edited for readability without altering their meaning. All interviews were analyzed using content analysis following Mayring [30] with MAXQDA 2022 (version 22.8.0; VERBI GmbH). Overall, 59 complete interviews and 2 summaries of interviews (where recording had not worked) were included in the analysis. Initially, 4 broad top-level codes were defined to classify the interview content according to the 4 constructs of technology acceptance. In the next step, potential subcodes were delineated on the basis of the interview guide. Subsequently, the interviews were analyzed using these preexisting codes, with the additional step of using an inductive method to derive new codes. Applying this method to both apps resulted in comparable coding systems that still account for individual differences. The resulting code system is illustrated in Figure 2.

While the UTAUT model was used as a foundation for both the interview guideline and the analysis, it was slightly adapted to fit this study. As participants in a medical study, users did not expect the devices to be personally useful and were unable to judge their usefulness to the study team. In addition, as we interviewed participants after their use of the device, “expectation” seemed to be an inaccurate term in this case. Therefore, we decided to use the terms “performance” and “effort or ease of use” instead of performance expectancy and effort expectancy, respectively.

### Participants

Interviews were conducted separately for each participant group until data saturation was reached. To ensure a diverse sample, we conducted at least 1 interview per patient group and research center. When new interviews in a patient group did not lead to new valuable insights, we assumed data saturation. This approach led to different sample sizes. A total of 61 interviews were conducted. As only 1 patient with systemic lupus erythematosus could be interviewed and therefore data saturation is not clear, this interview was excluded from analyses.

### Ethical Considerations

Ethics approval was granted by the ethics committee of Kiel University, Germany (Ethikkommission der Medizinischen Fakultät der CAU zu Kiel, approval D491/20) and the Health Research Authority and Health and Care Research Wales (IRAS project ID 282329, REC reference 20/PR/0185). Participants gave written and verbal informed consent before taking part in the study. Interviews were voluntary for participants of the larger FS. To ensure participant privacy, participants were assigned a random code. The code key is stored, encrypted, and password-protected. Interview transcripts were pseudonymized. Voice and video recordings are stored separately in accordance with the General Data Protection Regulation.

## Results

### Overview

Table 1 provides an overview of the number of interviews per group and site, while Table 2 shows the demographic characteristics of the sample.

**Table 1:** Number of interviews per group and location.

Location	Healthy participant, n (%)	PD^a^, n (%)	HD^b^, n (%)	SLE^c^, n (%)	PSS^d^, n (%)	RA^e^, n (%)	IBD^f^, n (%)
Kiel	11 (18.03)	12 (19.67)	—^g^	1 (1.64)	—	5 (8.20)	—
Münster	6 (9.84)	—	4(6.56)	—	—	—	—
Newcastle	2 (3.28)	—	—	—	11 (18.03)	1 (1.64)	—
Rotterdam	2 (3.28)	—	—	—	—	—	6 (9.84)

^a^PD: Parkinson disease.

^b^HD: Huntington disease.

^c^SLE: systemic lupus erythematosus.

^d^PSS: primary Sjögren syndrome.

^e^RA: rheumatoid arthritis.

^f^IBD: inflammatory bowel disease.

^g^Not applicable.

**Table 2:** Demographic data of the interview participants.

Group	Participants, n	Age (years), range	Sex			Living situation	
			Male, n (%)	Female, n (%)	Alone, n (%)		Not alone, n (%)
Healthy participant	21 (34.43)	21-77	12 (46.15)	9 (25.71)	7 (53.85)		14 (29.17)
PD^a^	12 (19.67)	37-80	8 (30.77)	4 (11.43)	1 (7.69)		11 (22.92)
HD^b^	4 (6.56)	29-59	2 (7.69)	2 (5.71)	1 (7.69)		3 (6.25)
PSS^c^	11 (18.03)	52-82	2 (7.69)	9 (25.71)	2 (15.38)		9 (18.75)
SLE^d^	1 (1.64)	53-53	0 (0)	1 (2.86)	0 (0)		1 (2.08)
RA^e^	6 (9.84)	40-68	0 (0)	6 (17.14)	1 (7.69)		5 (10.42)
IBD^f^	6 (9.84)	22-53	2 (7.69)	4 (11.43)	1 (7.69)		5 (10.42)
Total	61 (100)	21-82	26 (100)	35 (100)	13 (100)		48 (100)

^a^PD: Parkinson disease.

^b^HD: Huntington disease.

^c^PSS: primary Sjögren syndrome.

^d^SLE: systemic lupus erythematosus.

^e^RA: rheumatoid arthritis.

^f^IBD: inflammatory bowel disease.

Figure 2 shows the general code system used for both apps. Some of the second- and third-level codes were only used for one of the apps. *Quality of the questions*, *quality of speech function and tests*, as well as *usability of the reminder function* and *reactions of peers* were only needed for the symptom tracking app, while *feedback* was only used for the cognitive test app.

### Symptom Tracking App

#### Overview

Most participants used the symptom tracking app throughout the whole study period, though many participants mentioned missing several appointments. Three participants did not use the app at all. For one participant, it did not work, and some participants only got the notifications a few times for unknown technical reasons. One participant dropped out of the study due to perceived distress caused by the questions.

#### Performance

While most users rated the quality of the questions specially designed for the observational FS study as “fine,” there were some complaints. A few participants said that they had problems rating their experiences, and others criticized the questions as vague. A few users felt that there were not enough possibilities to answer, especially an option to answer “not at all” was missed. Some users felt that the questions did not fit their symptoms or experiences. In addition, a few participants were unsure about the meaning of some words: “I did not know, what Fatigue is. [...] And after I googled it, I was still unsure whether I had answered it correctly” (translated from German). One participant felt burdened by answering the questions:

At some point I said I didn’t want these questions anymore, I felt really bad about it. So for me, such questions don’t work, they also have to be positive. It’s a bit of a self-fulfilling prophecy, in that I always ask [myself] negative questions. So, I really found that I felt really, really bad afterwards.

Translated from German

Many participants found the questions boring or not well-suited to their situation, as they experienced little variation in their symptoms.

Some participants reported not liking the speech function researchers designed for the observational FS study, as they were unsure what to talk about or were embarrassed talking about their day: “I didn’t know exactly how precise I was supposed to talk about that” (translated from German). Other participants reported feeling silly while performing the speech tests:

The only thing I didn’t quite understand was that in the evening you either had to say “papapam” all the time or say “A” for a very long time and I didn’t quite understand the point of that, but I’m sure there’s a reason for that. [...] I just thought it was a bit silly.

Translated from Dutch

Some users pointed out that it was hard to stay motivated to answer the questions: “They were virtually the same questions. You just got fed up with them.” On the other hand, some users reported that they enjoyed monitoring their own data:

Comparing the beginning of the day to the end of the day [...]. And then looking at how I slept the night before. So, yeah, I enjoyed doing that part.

A few participants reported that they benefited from using the app, as it raised their awareness of their feelings and symptoms: “I found that very informative to me, to actually stop and think [...]. It was thought-provoking.” Another participant said:

I actually didn’t think the questions were bad at all, because you first had to deal with the question, ‘How am I actually doing in this area today?’ at that moment. And I also noticed how it changes during the day. When you are tired in the evening, you click on completely different values than in the morning when you have had a good night’s sleep.

Translated from German

#### Effort or Ease of Use

Many participants agreed that answering the questions was quickly done, though some still perceived the effort as high. This was mainly due to the number of times it came as a part of study design or because they had to keep 2 phones with them: “If I were going out, having to take two phones out with me [...] was a bit of a nuisance.”

While many users were comfortable with the apps’ use frequency, a lot of participants found it too frequent. Many users were unable to respond consistently, and some described the timing of the app interactions as incompatible with their daily schedules: “It’s a burden for the fixed time that’s involved in those things. I’ve missed [answering the questions] a number of times, because you’re in a meeting or whatever” (translated from Dutch), reported one participant.

Using the app was described as easy by most participants. Some users reported problems with opening the app, which could only be done using the notifications on the phone: “That is a bit hidden. You have to scroll down first. I actually didn’t find it at first” (translated from German), reported one participant.

A few users reported problems with setting the time with a dial. Most reported not using the reminder function, though some found it quite useful. Some mentioned that remembering to answer the questions was bothersome. Others felt bothered by the reminder.

While most participants did not feel disturbed by using the app, some reported that it disrupted their schedules. “To a certain extent, it almost drove your day,” reported one user. It was perceived as more disturbing at work, as there was not always time to use the app: “When I’m at work, in the car or on my bike, I can’t just open the app and [answer the questions], so sometimes you get [...] a kind of guilty feeling, those kinds of negative feelings” (translated from Dutch).

There were some technical problems reported with the symptom tracking app. Some participants received the notifications not often enough or not for the whole period—not knowing why. Two users reported not getting any notifications at all. Others reported the questions to be in a mix of English and German: “The questions [were] only in English, it took a few days until we had it [in German]” (translated from German).

#### Social Influence

While no participants reported concerns regarding reactions by their peers, many said that they avoided answering questions when in company, running errands, or working: “When you are in the car or somewhere in a store, you can’t just start taking your phone out” (translated from German). There were no reports about concerns regarding data privacy.

#### Facilitating Conditions

There were some insecurities mentioned regarding the symptom tracking app. As mentioned in the performance section, some users were unsure what to include during the speech function. Some participants were unsure how long after the scheduled time point they could still respond, which led them to miss some questions. A few suggestions to improve the study design and app use were to personalize the timing of the notifications, increase the time frame, or indicate how long the time frames were. Many participants would have preferred getting the questions less often. Others said that there should be the possibility to go back to correct their inputs. While some users said that the app could be used following the same schedule for a longer time, others said that they would not like that, or that it would only fit for people with more severe symptoms.

### Cognitive Test App

#### Overview

Most participants used the cognitive test app, though some users stopped during the NBX. Others reported that they did not always have time for the tasks. One participant did not use the app.

#### Performance

Regarding the PVT and DSST, most participants felt positive and motivated. “I enjoyed those little quiz things,” reported one user. A few participants disliked the PVT and described it as boring: “The first one got a bit laborious because you’re doing the same thing all the time.” In contrast, the NBX was disliked by most participants. Many found it too hard, which led to frustration or annoyance. “I just said, I got two right, I thought they’d be thinking I’m the village idiot here. [...] The [NBX] was by far the most frustrating,” said one participant. They reported feeling unmotivated, as they did not see any improvement: “But to actually not feel as if [...] this was really achieving anything. You got to the stage sometimes where you just said, well, I’m just going to press a button.” Only a few participants reported liking this task, seeing it as challenging or fun.

Some participants commented that they would have liked feedback on their performance, which might have improved motivation: “If it would show afterwards whether you had gotten better or worse, or whether that was depending on the time of the day, that would have interested me” (translated from German).

A few participants reported feeling like they benefited from the tasks and could use them for training and getting better. Others reported not feeling any training effect: “I’m not under the impression that I got faster or more experienced [...]. Consistent, probably age-appropriate, I think” (translated from German).

#### Effort or Ease of Use

Most participants agreed that it was not too much effort, though some said that the tutorial for new tasks was too long. While most participants agreed that the frequency of using the app chosen for the study was appropriate, some reported to have missed a few tests. Other users mentioned that the times were not fitting to their schedules.

Participants generally found the app easy to use. They liked the design as a chat: “It’s a nice way of being greeted: ‘Hello, are you ready for your next exam,’ and concentrate, and off you go. That’s all you have to do,” said one user.

Some participants reported disruptions to their daily routines, as they sometimes had difficulty fitting the app’s scheduled assessments into their workday, especially when tasks required concentration: “You really have to go into the corner, because during the day, when you’re at work, you can’t really do it” (translated from German).

Some technical problems with the app were mentioned. For a few participants, the notifications did not come regularly twice a day, but only once or only at the beginning of their trial period. In other cases, not all tasks were available. A few users had problems solving the tasks because another app notification covered a part of their screen.

#### Social Influence

No participant reported having concerns regarding data privacy. One user stated:

That sort of thing crosses your mind but obviously, the paperwork you’re given beforehand tells you how it’s going to be stored, who will have access, whether it’s being anonymized. [...] If I participate in some sort of medical research then [...] I expect it to be absolutely fine.

Nobody mentioned any reaction of peers.

#### Facilitating Conditions

Some participants reported feeling insecure whether they understood the instructions for the third task correctly. While some participants found the instructions sufficient, others felt that the instructions for the last task were vague. Regarding optimization, a few suggestions were made. Some participants suggested to make the NBX easier. Others reported wanting more variety in the tasks: “I would like to have more variety. So, maybe today like this and tomorrow like this, or maybe sometimes more difficult and sometimes easier, so that you are a bit [more] motivated” (translated from German), suggested one participant. While some study participants did not want to use the cognitive test app longer following this study-specific schedule, others reported that they would have liked to do more tasks.

## Discussion

### Principal Findings

This study considers the technology acceptance of 2 different apps in the context of the IDEA-FAST FS. We interviewed participants who used a symptom and activity app as well as a cognitive test app over 4 weeks. Using the UTAUT [13] model, we identified different themes of technology acceptance in the use of these apps.

For the users, performance played a less crucial role in technology acceptance, whereas the aspect of effort gained importance. Both facilitating conditions as well as social influence seemed to play minor roles in the acceptance of the apps.

### Performance

When users expect an mHealth app to be helpful to them, they show more willingness to use it [1,6,16]. This perceived usefulness has been shown to be a good predictor for the intention to use mHealth apps [17,18,21,23]. In this study, the apps did not provide an apparent impact on the participants’ health. Therefore, motivation and adherence to the schedule were issues with both apps. Some participants did not see value in answering the questions of the symptom tracking app and saw it as a burden, while others enjoyed the opportunity to track their behavior and symptoms. The cognitive test app varied in popularity among participants. Some users found the PVT boring, but it was well liked by most, as was the DSST. However, many participants disliked the NBX, found it too hard or frustrating. This led to compliance problems, such as randomly clicking through the test, ignoring or missing scheduled sessions, or ceasing to answer it altogether. A possible reason for this could be that the task was developed for healthy users. The cognitive impairments of participants with Parkinson disease and Huntington disease, along with the relatively high average age of the sample, contributed to the task being perceived as too difficult. As a result, the task was adjusted during the FS. However, since the adjusted NBX was still perceived as too difficult, it was not included in the subsequent clinical observation study.

An important aspect of motivation for participants seemed to be feedback on their data. While not many participants reported interest in the results from the symptom tracking app, those who enjoyed the app used it as a tool to track their behavior and symptoms, especially fatigue. Possibly, these were patients who were concerned about their fatigue before joining the study. In contrast, many participants voiced a wish for feedback from the cognitive tests. Feedback could help increase motivation if participants were able to see their performance over time. If an mHealth app itself does not seem inherently helpful to the user, feedback might serve as a useful way to engage them. At the same time, feedback could be considered as an intervention, which researchers might wish to avoid. Feedback can be a helpful or interesting resource users can gain from their app use.

### Effort or Ease of Use

People tend to use mHealth apps they perceive as easy to understand and to use. This effect has been shown in previous studies [1,6,16,18] and can be observed here as well. While users agreed that the apps did not take too much time, the frequency of use required by the study design was more problematic. Especially having to use the apps at specific time points was seen as disruptive and led to missing data. The 2 apps described in this study had to be used several times per day. Even though the sessions were short, these numerous sessions meant a considerable amount of extra effort, especially since the participants had to adhere to certain time constraints. It was not surprising that almost all participants reported they had not been able to attend all sessions, especially those who were more active or working. Participants suggested adjustable time frames as a solution to account for differences in daily routine or to find individual times they would be able to handle the apps, which should be considered by the clinical team to find a compromise between the patient’s needs and study goals. When choosing an app for a scientific study, it is beneficial if it is available for all common smartphone operating systems. Often, participants prefer using their own phone rather than a study phone. Study phones constitute an additional device participants have to familiarize themselves with, charge regularly, carry around, and keep in mind to use. They tend to be forgotten or ignored more easily.

### Social Influence and Facilitating Conditions

Both social influence and facilitating conditions did not seem to have significant impact on the acceptance of the apps in this study. An explanation could be the study context. As participants were using the apps without expected impact on their health, it is not surprising that family members and peers did not have specific expectations or motivating sentiments regarding app use. Therefore, opinions of peers seem to have had only a slight impact on their willingness to use the apps. This finding is not entirely inconsistent with the literature, as some studies have found no influence of social influence on the intention to use a technology [20,31]. Other studies have shown that opinions of peers influence the decision to use an mHealth app [21,23]. In the context of study-related apps, social influence may affect the decision to participate in the study rather than the use of the app itself. Another interesting finding was that participants did not voice major concerns regarding data privacy. Data privacy and security have previously been reported as a valid concern for users of mHealth apps [10,16,25,32], though others did not find that privacy concerns predicted intention to use mHealth apps [17,18,21]. As the symptom tracking app gathers quite personal information, concerns regarding data privacy had to be expected. When asked about data privacy, many participants cited trust in the study team and medical data security standards as reasons to feel safe.

Regarding facilitating conditions, salient factors such as costs for the use of mHealth apps or availability [16,23,33] were not an issue in this case. Both apps as well as study smartphones were provided for the participants. It seems that the provided support in the form of instructions and the study team was sufficient for most participants to feel confident about the correct use of the apps. There were some uncertainties concerning the symptom tracking app, like not knowing what to report using the speech function and not knowing how much time there was for answering the questions. Facilitating conditions have been found to be very influential on the intention of using a technology in several studies [1,33], while others did not find a connection [6]. It seems probable that due to the high amount of support and lack of limiting factors, the positive impact of facilitating conditions on the use of the apps was present. This assumption is supported by the fact that many participants stressed the good support during the study and voiced their appreciation for the study team.

Some aspects of these apps were controversial among participants. While many participants disliked the NBX of the cognitive test app, some voiced their appreciation for feeling challenged by the task. Many participants described the frequent documentation of their well-being as tedious, although some appreciated the possibility to track their symptoms. While studying the opinions and experiences of users is a valuable tool to ensure acceptance, users have different desires and needs. In choosing suitable mHealth apps, one size will most certainly not fit all. While some aspects of these apps are necessary for study success, such as the documentation in the symptom tracking app, others might be customizable. An example is the reminder function of the symptom tracking app, where users can decide for themselves if they want to be reminded or not.

### Limitations and Strengths

Limitations and strengths of this study align with those previously described [27]. In short, due to the design of the study, participants used multiple devices as well as the mHealth apps at the same time, which might have influenced their opinions. Due to the different number of interviews in each group, disease-specific aspects might not have been entirely captured. In addition, the COVID-19 pandemic might have had an influence on the acceptance of the 2 apps, since participants were partly living in lockdown or had altered their normal routines. We are also aware that these qualitative interviews primarily reflect app modifications made specifically for this FS, including support provided by the clinical team. Thus, the findings cannot be generalized to the assessment of app use in different contexts. The results represent the experiences of the FS participants with the apps implemented in a specific study with its own design choices. The requirements of apps can be different, depending on their context of use.

This study represents the opinions and voices of a large and diverse sample on the acceptance of 2 mHealth apps with different aims and uses. This enables us to draw conclusions, which might also be useful for other studies planning to use mHealth apps, as well as help in the development of new apps. Conducting these interviews during the duration of the FS helped us to improve and select suitable apps for use in the clinical observation study. As described earlier, the NBX of the cognitive test app was adjusted to fit better to the study population. Its use was discontinued for the clinical observation study. Our experience underlines the importance of involving users and patients in the development and design of mHealth apps in order to achieve a user-friendly and meaningful result [11].

### Recommendations

The choice of appropriate mHealth apps for a research project can be a challenging decision to make. The efficacy of any data collection app is contingent upon the extent to which its users actually use it. Consequently, when developing or selecting an mHealth app for a research project, it is essential to consider technology acceptance. The effort expended by the user should be considered as an integral component of the evaluation process. The ideal app is one that is straightforward and intuitive to use [24]. Patients will have divergent preferences regarding the impact of the study on their lives. Consequently, the integration of customizable features, where feasible, may enhance technology acceptance. These may encompass adjustable reminder functions, time points, and time frames for use of the app. The minimization of user effort is also relevant to the decision regarding the use of study phones. Many patients may prefer their personal smartphones as opposed to designated study phones. Therefore, ensuring that the app is available for all common smartphone operating systems might be beneficial [23]. The necessity of carrying 2 phones is thus eliminated, and the potential for patients to forget the phone at home and miss vital data is also minimized. Evidently, the time expended in using the app constitutes a significant proportion of the overall effort involved. Most cognitive testing and symptom tracking apps require patients to use them for a fixed period of time. Should this be required on a daily basis, or several times per day at designated times, it would necessitate a significant degree of effort. To minimize effort, it is vital to keep the required app time as short and infrequent as possible.

Considering the role of performance in technology acceptance, the pivotal question pertains to the extent to which the technology benefits the patient. The use of cognitive testing and symptom tracking apps within the context of research studies may not necessarily result in tangible health benefits for the patient. This is particularly relevant in cases where apps are used over an extended period of time. In such cases, consideration should be given to alternative factors of motivation. These elements may include gamification mechanisms or feedback on data in noninterventional studies. In the context of cognitive testing apps, the motivational factors of enjoyment and challenge can be leveraged. A task that is perceived as too easy may quickly become tedious to the user, while those that are too challenging may result in demotivation over time. The process of identifying an appropriate level of difficulty for the target population can be a difficult yet rewarding endeavor.

The role of social influence in the technology acceptance of mHealth apps in the context of medical studies may be less significant. It is possible that the opinion of significant others may influence patients’ decisions regarding participation in the study, thereby reducing the impact of social influence during the actual use of the apps. Data security could be a cause of concern for patients. The provision of information regarding data security measures during the recruitment process is therefore a valuable use of time [25].

Some salient factors that are typically considered facilitating conditions of technology acceptance, such as costs or availability [23], are typically negligible in the context of medical studies. Nevertheless, the pivotal role that technical and organizational support play in facilitating patient compliance has to be taken into account. The provision of adequate instructions and support should be considered a prerequisite [21]. The establishment of positive relations between patients and the study team can be a significant motivating factor, as well as enhancing satisfaction with the study itself.

### Conclusions

This study describes the technology acceptance of 2 mHealth apps. The primary objective of this study was to assess the usability of these apps in a follow-up investigation. As part of a larger evaluation study, potential issues were identified. They include demotivation due to the level of difficulty experienced with one of the cognitive tasks and challenges with adhering to the stipulated time schedule for the symptom tracking app. These issues were addressed through a variety of approaches. For instance, the challenging task was set aside for use in the follow-up. To support adherence, time points were limited to those essential for the study’s success while allowing as much flexibility as possible. The second objective of this study was to assess the specific challenges researchers encounter when selecting suitable mHealth apps for their own research, and what characteristics an app must possess to be considered a useful tool for health research. The incorporation of patient perspectives in the device selection process has been demonstrated to be beneficial and valuable. The findings of this study provide a valuable contribution to the field of medical research by offering insights that may inform the selection of appropriate mHealth apps. The study emphasizes the importance of effective communication and testing of all materials used in conjunction with patients and intended users prior to the commencement of research studies.

## Abbreviations

DSST: digit symbol substitution test
FS: feasibility study
IDEA-FAST: Identifying Digital Endpoints to Assess Fatigue, Sleep and Activities of Daily Living in Neurodegenerative Disorders and Immune-Mediated Inflammatory Diseases
mHealth: mobile health
NBX: n-back task
PVT: psychomotor vigilance task
UTAUT: unified theory of acceptance and use of technology
